# Evaluation of 10 AMD Associated Polymorphisms as a Cause of Choroidal Neovascularization in Highly Myopic Eyes

**DOI:** 10.1371/journal.pone.0162296

**Published:** 2016-09-19

**Authors:** Alvaro Velazquez-Villoria, Sergio Recalde, Jaouad Anter, Jaione Bezunartea, Maria Hernandez-Sanchez, Laura García-García, Elena Alonso, Jose María Ruiz-Moreno, Javier Araiz-Iribarren, Patricia Fernandez-Robredo, Alfredo García-Layana

**Affiliations:** 1 Ophthalmology Experimental Laboratory, Universidad de Navarra, Pamplona, Spain; 2 Department of Ophthalmology, Clínica Universidad de Navarra, Pamplona, Spain; 3 Department of Celular and Molecular Medicine, Centro de Investigaciones Biológicas and Ciber de Enfermedades Raras, Madrid, Spain; 4 Department of Ophthalmology, Castilla La Mancha University, Albacete and Baviera European Institute of Retina, Alicante, Spain; 5 University of the Basque Country (Surgical-Clinical Institute of Ophthalmology) and San Eloy Hospital, Bilbao, Spain; University of Cologne, GERMANY

## Abstract

Choroidal neovascularization (CNV) commonly occurs in age related macular degeneration and pathological myopia patients. In this study we conducted a case-control prospective study including 431 participants. The aim of this study was to determine the potential association between 10 single nucleotide polymorphisms (SNPs) located in 4 different genetic regions (*CFI*, *COL8A1*, *LIPC*, and *APOE)*, and choroidal neovascularization in age-related macular degeneration and the development of choroidal neovascularization in highly myopic eyes of a Caucasian population. Univariate and multivariate logistic regression analysis adjusted for age, sex and hypertension was performed for each allele, genotype and haplotype frequency analysis. We found that in the univariate analysis that both single-nucleotide polymorphisms in *COL8A1* gene (rs13095226 and rs669676) together with age, sex and hypertension were significantly associated with myopic CNV development in Spanish patients (p<0.05). After correcting for multiple testing none of the polymorphisms studied remained significantly associated with myopic CNV (p>0.05); however, analysis of the axial length between genotypes of rs13095226 revealed an important influence of COL8A1 in the development of CNV in high myopia. Furthermore we conducted a meta-analysis of *COL8A1*, *CFI* and *LIPC* genes SNPs (rs669676, rs10033900 and rs10468017) and found that only rs669676 of these SNPs were associated with high myopia neovascularization.

## Introduction

Myopia is the most common ocular disorder worldwide [1] with an approximate prevalence rate of 27% in the United States and Western Europe [2], and has increased in the last 50–60 years [3,4]. The prevalence of myopia in Asian populations is even higher [1,2,5–9]. In some East and Southeast Asian countries, nearly 80% of the population has myopia, whereas 10–20% has high myopia (HM) [10–12].

HM is defined as having an as axial length ≥26.5 mm or ≤−6 diopters (D) of myopic refractive error [13,14]. Myopic axial length elongation can appear as retinal pathological changes and ocular comorbidities that may lead to blinding disorders, including chorioretinal atrophy and choroidal neovascularization (CNV), premature cataracts, retinal detachment, and glaucoma. HM is a common vision-threatening disease that affects 0.5–5.0% of the population worldwide [1,3,15]. It is now considered the fourth-most common cause of irreversible blindness in developed countries after age-related macular degeneration (AMD), glaucoma, and diabetic retinopathy [16], leading to a substantial impact on the public health economy. Therefore, it is crucial to elucidate the pathological mechanisms underlying HM to develop new therapies for this disabling pathology.

Epidemiological studies have shown that both genetic and environmental factors contribute to myopia development [17–23]. However, after years of intensive research, the precise mechanisms controlling ocular growth and development of refractive errors remain unclear. Genetic associations with HM have been investigated for several decades. Twin and family studies have shown that myopia, particularly HM, has a high heritability [24,25]. It is now generally accepted that there are major genetic contributions to HM, in contrast non-pathological myopia appears to be multifactorial, possibly involving a large number of genes with small individual effects, and major environmental factors. Several HM susceptibility genes have been identified in linkage and candidate gene studies, such as paired box gene 6 (*PAX6*) [26], collagen type II alpha 1 (*COL2A1*) [27], collagen type I alpha 1 (*COL1A1*) [28,29], myocilin (*MYOC*) [30,31], hepatocyte growth factor (*HGF*) [32], transforming growth factor beta 1 (*TGFB1*) [33,34], transforming growth-induced factor (*TGIF*) [35,36], and lumican (*LUM*) [37,38].

CNV beneath the fovea is a common complication of HM and AMD and is a common cause of visual impairment [39]. Myopic CNV (mCNV) typically occurs in the fourth and fifth decades of life; HM is the most common cause of CNV in patients younger than 50 years of age. Moreover, CNV can develop in highly myopic eyes in the elderly population [40]; however, most highly myopic eyes never develop mCNV. In the elderly population, the most frequent cause of CNV is AMD. Both AMD and HM are caused by interactions between genetic and environmental factors, and genetic factors are thought to strongly contribute to CNV development in AMD patients [41–44]. Our previous studies [45]; suggested that the development of AMD-CNV may have a common genetic origin with mCNV, but we did not find a significant association between two-single nucleotide polymorphisms (SNPs) strongly associated with AMD (rs1061170 [Y402H] in *CFH* and rs10490924 [A69S] in *ARMS2*) and myopic CNV in a Spanish population. These results were confirmed in Japanese population by Nakanishi et al [46], who found no association between mCNV development and the three most important SNPs associated with AMD development in the Japanese population (rs1061170 (Y402H), rs10490924 (A69S) and rs11200638 of HTRA1). More recently, Leveziel et al [47] conducted a case-control study analyzing 15 genes associated with AMD in a North American Caucasian population of European origin, and found that only one SNP located in the gene for the complement factor I *(CFI)* significantly associated with mCNV. Despite the similarities between wet AMD and mCNV in the growth of macular new microvasculature in the choroid layer, only this SNP involved in the pathogenesis of wet AMD was previously reported to be associated with mCNV development.

Because the prevalence of HM varies among the populations, there may also be differences between the genetic variants associated with CNV, and potential associations in other races and other SNPs recently found to be associated with AMD require further analysis. The purpose of this study was to determine the contribution of some of the most important CNV-AMD-associated SNPs in a Spanish population and several recently described AMD-associated SNPs located in 4 genetic regions (*COL8A1*, *LIPC*, *CFI and APOE*) to the development of mCNV in a Spanish population.

## Materials and Methods

### Study subjects

Two hundred fifty unrelated highly myopic Spanish Caucasian patients (147 HM patients with mCNV, 103 HM patients without mCNV (HMnoCNV) patients) and 181 controls were recruited from various centers of the Red Temática de Investigación Cooperativa (RETICS). The inclusion criteria for the HM group were spherical refractive error ≤ -6.00 diopters (D) or an axial length ≥ 26 mm, and age over 50 years at the time of inclusion in the study. HM patients were divided into two groups: one group with mCNV in at least 1 eye and the other group without mCNV, who did not present any chorioretinal degeneration and had a visual acuity over 20/32 in both eyes. The population based control group was composed of patients aged over 50 years with spherical refractive error > -6.00 D without any pathology in the retina who came to annual revision. The exclusion criteria were tractional maculopathy, and/or epiretinal membrane in the OCT or other diseases different from HM that may occur with CNV (e.g. wet AMD, diabetic retinopathy, pseudoxantoma elasticum, presumed ocular histoplasmosis syndrome, lacquer cracks due to trauma). Patients presenting macular drusen or patients with a family history of AMD, known genetic diseases associated with myopia, such as Stickler or Marfan syndrome, and any ocular surgery, were also excluded, with the exception of cataract surgery.

All procedures used in this study conformed to the guidelines of the Declaration of Helsinki. The Institutional Review Board and the Ethics Committee of Clínica Universidad de Navarra approved the protocols used in this study. All patients were fully informed of the purpose and procedures, and written consent was obtained from each patient. All cases underwent detailed ophthalmologic examination, including visual acuity assessment, dilated slit-lamp biomicroscopy, automatic objective refraction, measurement of the axial length by A-scan ultrasound (UD-6000, Tomey, Nagoya, Japan) or partial coherence interferometry (IOLMaster, Carl Zeiss Meditec, Jena, Germany), color fundus photography, optical coherence tomography, and fluorescein angiography. Controls underwent visual acuity assessment, mydriasis fundus examination, and measurement of refractive error and axial length.

### Genotyping

We examined the available literature regarding genetic associations in AMD and the SNPs showing the most significant associations were selected. Genomic DNA was extracted from oral swabs using QIAcube (Qiagen, Hilden, Germany) and processed in the Laboratory of Experimental Ophthalmology at the University of Navarra (Spain). A set of 10 SNPs in 4 previously identified AMD-associated genes (shown in S1 Table) were genotyped in the control, HMnoCNV and mCNV cohorts using genomic DNA by allelic discrimination with validated assays (TaqMan; Applied Biosystems, Foster City, CA, USA; S1 Table) and real-time PCR (PE7300; Applied Biosystems), according to the manufacturer's instructions. None of the SNPs were in linkage disequilibrium except the SNPs in *CFI*, which have been classified as risk or protective haplotypes [48,49]; therefore the haplotypes of this gene were analyzed.

### Literature search strategy for meta-analysis

**Fig 1:**
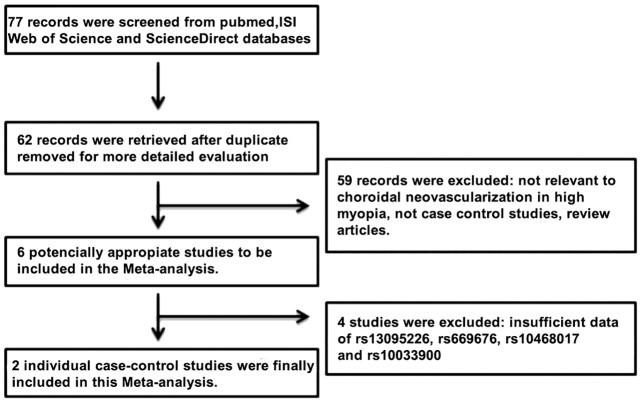
Flow chart for the selection of studies according to the criteria of this Meta-analysis.

For the comparative study between our results and previous studies related with mCNV, we conducted a meta-analysis of principal SNPs analyzed in this study. Potential articles were identified by a systematic search of the ISI Web of Science, PubMed, and ScienceDirect databases through June 09, 2016 using combinations of the following search terms: "*collagen type VIII alpha 1*" OR " *COL8A1*", "polymorphism" OR "SNP" AND "CNV High myopia" without language or publication date restrictions. All relevant publications and their reference were manually screened to identify eligible studies (Fig 1). Selection criteria papers identified during the literature search had to meet the following inclusion criteria for inclusion in our study: 1) case-control or cohort design studies of humans; 2) evaluation of the association of *COL8A1*, *CFI* and *LIPC* genes polymorphisms (rs13095226, rs669676, rs10033900, and rs10468017) with neovascularization high myopia; 3) sufficient published data available for our team to estimate the odds ratios (ORs) of different genotype frequencies; and 4) published original full-text literature. Studies meeting the following criteria were excluded from the analysis: 1) insufficient reported data; 2) abstracts, review papers, and case-only studies; 3) duplication of previously published literature. Three investigators (Velazquez, Fernandez-Robredo, and Recalde) independently extracted the data. Discrepancies between different investigators were resolved by 2 other investigators (Garcia and Hernandez) to reach a consensus. The collected data included: name of the first author, publication date, geographical location, numbers of cases and controls, ethnicity, genotyping methods and matching variables (Table 1).

**Table 1:** Main characteristics of eligible studies included in the Meta-analysis.

First Author	Year	mCNVn	Controlsn	Region	Ethnicity	Genotyping Method	Matching
Leveziel et al. [47]	2012	71	196	France/USA	Caucasian	ABI	Sex and Ethnicity
Miyake et al.[50]	2013	478	557	Japan	Asian	ABI	Age, sex, and axial length
Velazquez Villoria et al.	2016	147	103	Spain	Caucasian	TaqMan	Age,sex and ethnicity

### Statistical analyses

General characteristics (age, gender, refractive error, tobacco, HT and hypercholesterolemia) of patients with HM (mCNV and HMnoCNV) and controls were tested using Student´s *t-*test for the continuous variables age and refractive error, or chi-square test for the categorical variables sex, HTA, HC, and tobacco history. The frequencies of alleles, genotypes, and haplotypes were calculated in all the groups and were compared using a chi-square test and Fisher’s exact test and corresponding ORs were calculated. All SNPs analyzed in this study were in Hardy-Weinberg equilibrium (S1 Table).

Univariable logistic regression adjusted for all covariates was used to estimate the ORs and 95% confidence intervals (95% CI) using SNPStats software (Sole X et al., 2006). Analyses were performed for each genetic variant independent of other variants using codominant, dominant, recessive, and/or overdominant genetic models in base Akaike information, which chooses the inheritance model that best fits the data. We also used a multivariate logistic regression model to take into account all SNPs studied and environmental variables. Correction for multiple testing was performed using the Bonferroni method. For SNPs showing a significant association (rs13095226 and rs669676), to verify the influence of genotype on ophthalmological variables (refractive power and axial length), independent regression analysis was performed. Statistical analysis was conducted with SPSS 20.1 Software (SPSS Inc., Chicago, IL, USA). For all statistical tests, corrected p values of < 0.05 (two-tailed) were considered statistically significant. Separate meta-analyses were conducted on each of the 3 polymorphisms (rs669676, rs10033900 and rs10468017) determined from the articles during the systematic review. We used Z test to judge the significance of the pooled ORs, and statistical significance was considered when p < 0.05. Heterogeneity among included studies was evaluated by the 2-based test and the 2 index; < 0.10 or 2 > 50% were considered statistically significant. The random effects model was used to estimate pooled ORs when obvious heterogeneity was present; otherwise the fixed-effects model was used. Significant publication bias was considered when p < 0.05. All statistical analyses were performed using STATA 12.0 software (StataCorp LP, College Station, TX, USA).

## Results

The demographic characteristics of the study population are shown in Table 2. The mCNV group showed no significant differences in age, gender, refractive error, axial length, tobacco history, and hypertension with respect to the HMnoCNV group. Compared to the control group, the mCNV group showed significant differences in gender (p < 0.05), very significant differences in hypertension (p < 0.01), and highly significant differences in age, axial length, and refractive power (p < 0.001).

**Table 2:** Characteristics of the study population.

	High myopia		P-value 1	control group	P-value 2
	mCNV	HMnoCNV		No CNVNo HM-AMD	
Number of patients	147	103		181	
Age (Mean ± SD)	60.68 (±14.07)	58.58 (±13.85)	ns	73.39 (±5.31)	***
Female Gender %	67.35%	69.90%	ns	53.59%	*
Axial Lenght (mm ± SD)	30.74 (±2.60)	29.81 (±2.61)	ns	23.46 (±0.49)	***
Refractive power (Diopters ± SD)	-13.30D (±4.25D)	-12.84 (±4.21D)	ns	-1.77 (±0.93D)	***
Tobacco smokers	31.29%	27.22%	ns	23.76%	ns
Hypertension %	29.25%	23.33%	ns	46.96%	**

CNV, Choroidal Neovascularization; NA, not available; SD, Standard deviation.

^1)^ P-value comparing HM-CNV+ with HM-CNV-

^2)^ P-value comparing HM-CNV+ with Population based control group.

Significance:

ns p > 0.05;

* p < 0.05;

** p < 0.01;

***p < 0.001.

Tables 3 and 4 show the allele and genotype frequencies, ORs, p-values, and corrected p-values for the candidate SNPs, associations with the mCNV vs HMnoCNV group and vs control population. Univariate logistic regression analyses results were adjusted for age, gender, and HT. The SNPs rs13095226 and rs669676 in *COL8A1* showed a significant association with mCNV. In SNP rs13095226, the C allele showed a significant difference between mCNV patients and the HMnoCNV group [p = 0.034, OR = 2.0 (1.1–3.9)] but was no longer significant after Bonferroni correction was performed (p = 0.30). Genotype analysis showed also significant results in rs13095226 in the mCNV vs HMnoCNV groups in the recessive model [p = 0.0097, OR NA (0.0-NA)], but were no longer significant after multiple testing correction (p = 0.08). Furthermore, when mCNV patients were compared with the population-based control group (Table 4), both SNPs (rs13095226 and rs669676) were significantly associated with high myopia, but no longer significant when multiple testing corrections were performed. For the SNP rs669676, the recessive model of genotype analysis, the minor allele homozygotes (AA versus GG-GA), showed significant differences in the mCNV group vs controls without the HM group [p = 0.023, OR = 2.48 (1.12–5.49); however, Bonferroni correction revealed no association (p corrected = 0.20). Similarly, for rs13095226, allele C was related to a significant risk of mCNV vs. in controls [p uncorrected = 0.016; OR = 2.2 (1.2–4.2); p corrected = 0.14]. Genotype analysis also showed significant results for the TT-CT genotypes versus CC, [p uncorrected = 0.0096 and OR = NA (0.0–NA) p corrected = 0.08]. The remaining SNPs evaluated did not show a significant association with mCNV after adjusting for confounding factors.

**Table 3:** Comparison of the genotype frequencies between mCNV and HMnoCNV group.

SNPs	Gene	Chr	Genotype	HM mCNV Genotype freq (%)	MAF	HMnoCNV Genotype freq (%)	MAF	Allele *Uncorrected P-value	Allele OR (95% CI)	Allele **Corrected P-value	Genetic model Co/Do/Re/Ov	Gen *Uncorrected P-value	Genotype OR (95% CI)	Gen **Corrected P-value
**rs11728699** [48]	*CFI*	Chr4	GG/GT/TT	24.1/48.1/27.8	0.48	32.6/44.2/23.3	0.55	0.20	0.8 (0.5–1.1)	-	Do	0.13	1.66 (0.87–3.18)	
**rs13117504** [48]	*CFI*	Chr4	CC/CG/GG	28.8/44.6/26.6	0.49	21.1/48.9/30.0	0.54	0.25	0.8 (0.5–1.1)	-	Re	0.13	1.66 (0.85–3.23)	
**rs10033900** [48]	*CFI*	Chr4	CC/CT/TT	35.2/46.0/18.7	0.42	41.1/45.6/13.3	0.36	0.24	1.3 (0.7–2.2)	-	Re	0.26	1.57 (0.71–3.48)	
**rs11726949** [48]	*CFI*	Chr4	CC/CT/TT	90.7/8.5/0.8	0.05	86.5/12.4/1.1	0.07	0.41	0.7 (0.3–2.2)	-	Do	0.47	0.72 (0.30–1.76)	
**rs6854876** [48]	*CFI*	Chr4	GG/CG/CC	29.3/47.4/23.3	0.47	23.5/44.7/31.8	0.54	0.17	0.7 (0.5–1.3)	-	Re	0.12	0.60 (0.31–1.15)	
**rs7439493** [48]	*CFI*	Chr4	GG/GA/AA	28.6/45.1/26.3	0.49	22.4/45.9/31.7	0.55	0.24	0.8 (0.5–1.1)	-	Re	0.19	1.56 (0.80–3.05)	
**rs13095226** [47,51]	*COL8A1*	Chr3	TT/CT/CC	77.8/17.0/5.2	0.14	85.6/14.4/0.0	0.07	0.034	2.0 (1.1–3.9)	0.30	Re	0.0097	NA (0.00-NA)	0.08
**rs669676**[47,51]	*COL8A1*	Chr3	GG/GA/AA	35.5/40.3/24.2	0.44	30.4/54.4/14.2	0.41	0.69	1.1 (0.7–1.9)	-	Ov	0.15	0.66 (0.37–1.17)	
**rs10468017** [52]	*LIPC*	Chr15	CC/CT/TT	46.6/42.1/11.3	0.32	49.4/40.2/10.3	0.30	0.75	1.1 (0.6–2.0)	-	Co	1	1.01 (0.37–2.73)	
rs769455*** [53]	*APOE*	Chr19	CC	100.0/0.0/0.0	-	100.0/0.0/0.0	-	-	NA	-	-	-	**-**	

Chr: Chromosome; Genotype freq: Genotype frequency; MAF: minor allele frequency, OR: Odds ratio; CI: Confidence interval; Co: Codominat; Do: Dominant; Re: Recessive; Ov: Overdominant;

*Uncorrected P-value: value from logistic regression model; P value significance < 0.05;

**P-value corrected for multiple testing using Bonferroni method.

***rs769455 was excluded from the analysis because it was monomorphic SNP.

**Table 4:** Comparison of the genotype frequencies between mCNV and population based control group.

SNPs	HM mCNV Genotype freq (%)	MAF	control group Genotype freq (%)	MAF	Allele *Uncorrected P-value	Allele OR (95% CI)	Allele **Corrected P-value	Genetic model Co/Do/Re/Ov	Gen *Uncorrected P-value	Gen OR (95% CI)	Gen **Corrected P-value
**rs11728699**	24.1/48.1/27.8	0.48	24.7/51.7/23.6	0.50	0.78	0.9 (0.6–1.3)		Re	0.41	1.34 (0.66–2.71)	-
**rs13117504**	28.8/44.6/26.6	0.49	22.7/58.0/19.3	0.48	0.53	1.0 (0.6–1.8)		Re	0.072	1.92 (0.94–3.91)	-
**rs10033900**	35.2/46.0/18.7	0.42	30.2/52.1/17.6	0.43	0.65	0.9 (0.5–1.6)		Ov	0.25	0.70 (0.38–1.29)	-
**rs11726949**	90.7/8.5/0.8	0.05	88.2/11.8/0.0	0.06	0.70	0.8 (0.4–2.0)		Do	0.12	0.43 (0.14–1.26)	-
**rs6854876**	29.3/47.4/23.3	0.47	24.1/50.9/25.0	0.50	0.47	0.9 (0.6–1.3)		Co	0.79	1.32 (0.56–3.11)	-
**rs7439493**	28.6/45.1/26.3	0.49	24.1/57.8/18.1	0.47	0.37	1.1 (0.7–1.6)		Co	0.13	2.06 (0.84–5.03)	-
**rs13095226**	77.8/17.0/5.2	0.14	86.7/13.3/0.0	0.07	0.016	2.2 (1.2–4.2)	0.14	Re	0.0096	NA (0.00-NA)	0.08
**rs669676**	35.5/40.3/24.2	0.44	34.9/48.6/16.5	0.41	0.25	1.1 (0.8–1.7)		Re	0.023	2.48 (1.12–5.43)	0.20
**rs10468017**	46.6/42.1/11.3	0.32	50.6/33.1/16.3	0.33	0.93	0.9 (0.6–1.4)		Ov	0.063	1.15 (0.65–2.05)	-
rs769455***	100/0.0/0.0	-	100/0.0/0.0	-	SD	-	-	Co	0.68	1.45 (0.61–3.44)	-

Genotype freq: Genotype frequency; MAF: minor allele frequency, OR: Odds ratio; CI: Confidence interval; Co: Codominat; Do: Dominant; Re: Recessive; Ov: Overdominant;

*Uncorrected P-value: value from logistic regression model; P value significance < 0.05;

**P-value corrected for multiple testing using Bonferroni method.

***rs769455 was excluded from the analysis because it was monomorphic SNP.

rs13095226 was not included in the multivariate analysis because none of the HMnoCNV participants showed the minor allele homozygote genotype (CC). Thus, only age and hypertension showed a significant association in the multivariable analysis between mCNV and HMnoCNV [p = 0.027 OR = 0.45 (0.22–0.91), p = 0.011 OR = 0.43 (0.22–0.82) respectively] (Table 5).

**Table 5:** Multivariable logistic regression analysis between mCNV and HMnoCNV.

	B	Sig.	OR	95% CI	
				Lower	Upper
mCNV vs HM No CNV					
Tobacco (1)	0.848	0.006	2.33	1.27	4.28
Age	-0.558	0.096	.57	.29	1.10

Reference: Good response.

B: multivariate logistic regression value, Sig: P-value significance (< 0.05), OR: Odds Ratio, CI 95%: 95% confidence interval.

**Fig 2:**
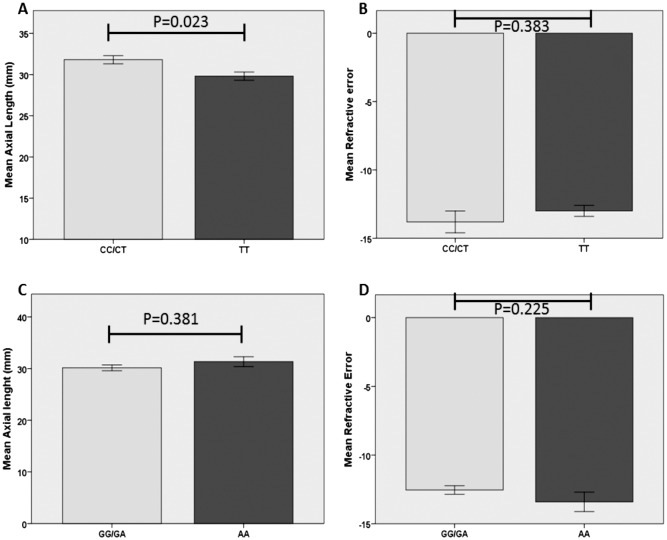
Axial length and refractive error of Col8A1 gene SNPs (rs13095226 and rs669676) genotypes. (A-B) Axial length showed statistically significant differences between CC/CT vs TT genotypes of rs13095226, whereas no differences were found for Refractive error. (C-D) Axial length and Refractive error (respectively) did not show differences between GG/GA vs AA genotypes of rs669676.

Based on the significant association of rs13095226 and rs669676 before applying Bonferroni correction, we independently analyzed the ophthalmological parameters refractive power and axial length to determine the influence of the genotype on the distribution of these variables (Fig 2). Our results showed a significant association (p = 0.023) between the CT/CC genotype of rs13095226 and a longer axial length (Fig 2A), but this association was not found with the refractive power (Fig 2B). rs669676 showed no significant association either with axial length or number of diopters (Fig 2C and 2D).

Furthermore, we evaluated the inferred haplotype frequencies in the CFI gene showing a strong association with AMD in previous studies between the mCNV and HMnoCNV groups, which are shown in Table 6. None of the haplotype frequencies were significantly different after multiple testing corrections. Haplotype analyses between the mCNV group and the population-based control group for the CFI gene showed no significant associations (data not shown).

**Table 6:** Haplotype analyses of LD blocks in the CFI gene.

SNPs	Haplotype	Freq mCNV	Freq HMnoCNV	P-Value	OR
rs11728699/rs6854876/rs7439493/rs13117504	GCAG	0.444	0.507		1.00
	TGGC	0.484	0.424	0.17	1.32 (0.89–1.97)
	GCGC	0.045	0.029	0.46	1.55 (0.48–5.01)
	TGAG	0.027	0.029	0.77	0.70 (0.05–9.05)
rs10033900/rs11726949	CC	0.568	0.614		1.00
	TC	0.356	0.382	0.35	1.22 (0.81–1.85)
	TT	0.045	0.035	0.8	0.86 (0.27–2.72)
	CT	0.015	0.024	0.78	0.79 (0.16–4.02)
rs13117504/rs10033900	GC	0.4412	0.501		1
	CT	0.370	0.331	0.32	1.25 (0.81–1.92)
	CC	0.139	0.137	0.49	1.23 (0.68–2.22)
	GT	0.049	0.031	0.72	1.23 (0.39–3.90)

Freq mCNV: Frequency of Myopic Choroidal Neovascularization, Freq HMnoCNV: Frequency of High Myopic eyes without Choroidal Neovascularization, P-value significance (<0.05), OR: Odds Ratio

**Fig 3:**
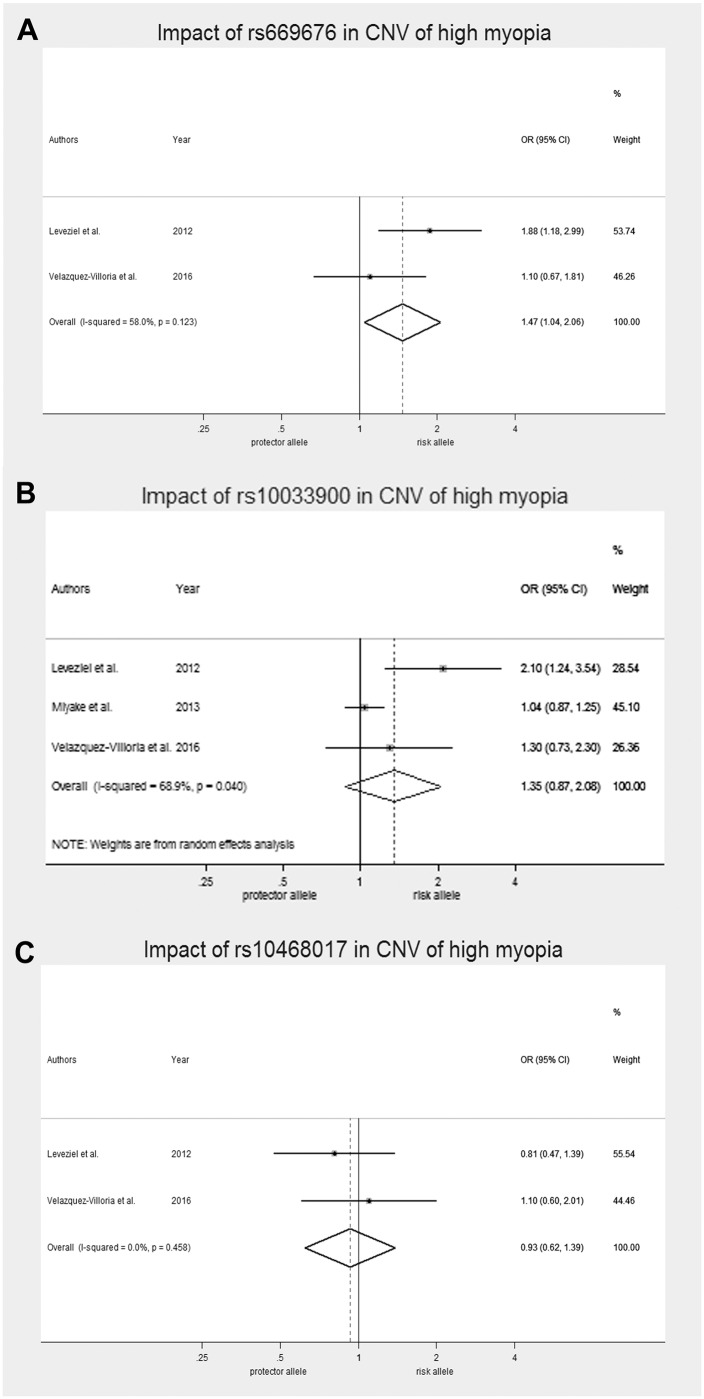
Forest plots of the association of polymorphisms and susceptibility to mCNV. A) Impact of rs669676 in mCNV. B) Impact of rs10033900. C) Impact of rs10468017.

We also conducted a meta-analysis of the 3 principal SNPs identified in previous studies (rs669676, rs10033900, and rs10468017). After systematic review, only two studies (Leveziel et al. [47] and Miyake et al. [50]) met the review requirements. A separate meta-analysis of these 3 SNPs revealed that only the rs669676 SNP presented significant association with mCNV (OR 1.47; IC95% 1.0–2.1) (Fig 3 and S2 Table). The rs10033900 showed high heterogeneity with I-squared values of 68.9% and we used the random-effect model to evaluate this SNP, while the fixed effect model was used for rs10468017 and rs669676.

## Discussion

CNV is a major cause of visual impairment in highly myopic patients and is the second leading cause of neovascular maculopathy after AMD. CNV is a common process in both diseases [39] and it can be difficult or impossible to distinguish between CNV resulting from excessive axial elongation and CNV attributable to the etiology of wet-type AMD in highly myopic eyes [39,54–56]. Thus, we hypothesized that SNPs associated with neovascularization in wet AMD may have be related to mCNV.

Our previous study [45] of *CFH* and *ARMS2* was conducted in a cohort > 30 years of age, with subjects showing no significant differences. Recent studies have suggested the importance of other genetic regions in the development of wet AMD. Thus, we evaluated 10 new AMD-associated SNPs located in 4 different genes (*COL8A1*, *LIPC*, *CFI and APOE*) in an older population.

Our results showed that only the *COL8A1* gene was significantly associated with mCNV in a Caucasian Spanish population before multiple analysis correction. In the SNP rs13095226, the C allele showed OR = 2.0 for developing CNV in Spanish high myopic patients. For SNP rs669676, the recessive model of genotype analysis, GG-GA versus AA showed similar risk ORs vs the control group (OR = 2.4). These results indicate that this gene is important in the development of myopic CNV. We verified the influence of this SNPs using quantitative ophthalmological variables. Our results showed that heterozygotes and minor allele homozygotes of rs13095226 (dominant model, CT/CC) had significantly higher axial length than patients presenting major allele homozygosis (TT). Furthermore our results showed that the rs13095226 genotype is associated with axial length (Fig 2A), but not with refractive power (Fig 2B). This may be because refractive power is influenced not only by the axial length of the eye, but also by the corneal keratometry and the lens. These results confirm the influence of this protein in axial growth and thus CNV development resulting from excessive axial elongation.

*COL8A1* encodes one of the two alpha chains of collagen type VIII, a major component of ocular basement membranes such as Bruch's membrane and choroidal stroma [57]. Collagen VIII is produced by muscle cells and macrophages in the vascular wall. This protein regulates matrix metalloproteinases activity and endothelial cell migration mediated by vascular endothelial growth factor during angiogenesis. Col8A1 protein may lead to direct or indirect structural alterations in the Bruch’s membrane, which is a risk factor for mCNV. [58] Numerous studies have investigated the association between alterations in genes encoding the different collagen types and the development of HM, finding discrepant results between different populations[59–64]. Furthermore, many of the syndromes caused by alterations in collagen are associated with the development of HM resulting from excessive axial elongation [65–69]. Taken together, our results and those obtained in other studies [44,47,51,70,71] indicate the importance of COL8A1 in retinal angiogenesis, both in pathological myopia and AMD. This may be related to alterations in *COL8A1* which can alter its expression to favor angiogenesis in these patients. This may be because proteins expressed by this gene stabilize the phenotype of endothelial cells [72]. During angiogenesis, where the differentiated phenotype is lost, the control and regulation of type VIII collagen may play a key role in processes in which new blood vessel formation is required and when excessive blood vessel formation is a pathological event (neovascularization).

Leveziel et al [47] previously studied the SNP rs669676 and only found a significant association with mCNV in multivariable analysis, which was no longer significant after correction for multiple testing. In our study, the recessive model of genotype analysis of the SNP rs669676 showed significant differences in mCNV vs controls without the HM group. Validating the results obtained by Leveziel et al, a comparison between highly myopic cohorts with or without CNV in rs669676 frequencies showed no statistically significant differences, but meta-analysis results of our study together with the Leveziel et al, showed a significant association of rs669676 with with mCNV. So, we confirmed a statistically significant association of the rs669676 validating the importance of the *COL8A1* gene in the development of mCNV.

One of the most important systems related to the development of wet AMD is the complement system. In this study, we analyzed complement factor I (*CFI*), which showed no association both individually and as *CFI* SNPs haplotypes. This may be because the complement system is more closely associated with the etiology of AMD and less with that of HM. In AMD, waste substances excessively accumulate between the layers of the retina, leading to chronic activation of the complement system [73]. However, HM is caused by excessive axial eye growth and does not appear to be related to chronic activation of the complement system as in AMD.

Moreover, the results of the studies that analyzing the association between *CFI* and mCNV have not been replicated. Leveziel et al reported an increased risk of mCNV associated with the T allele of rs10033900, which is located 2781 base pairs upstream of the 3´-untranslated region of the CFI gene, in a Caucasian US population with European ancestry [47]. However, Miyake et al found no association between this SNP and mCNV in a Japanese population [50]. Because the results of the previous studies were not consistent, we evaluated all haplotypes in the CFI gene previously found to be associated with CNV in AMD [48,74]. We found no association between this gene and mCNV.

Previous studies have not shown consistent results. We performed a meta-analysis based on previously published data, but found no significant associations. The large heterogeneity (I-squared 68.9%) observed in the meta-analysis can be explained by the different ethnic origins of the populations studied, which may have led to different responses of the SNPs. Additional studies are needed to elucidate the importance of these genes in the development of myopic CNV, particularly those focused on rs13095226 of *COL8A1*. This is the first study examining this SNP in mCNV; a previous study evaluated wet AMD and found no significant association in Chinese patients [75]. There are at least two main strengths to this study. First, all participants were from a similar ethnic background, reducing the heterogeneity in different populations. Second, the mCNV group was compared not only with HM without CNV patients, but also with a population-based control group. Both HM cohorts had a high myopic genetic profile; therefore, we compared these cohorts with a control group of very advanced age to rule out the genetic predisposition for developing CNV (both mCNV and AMD-CNV). However, there were some limitations to this study. First, the sample size was low, and no cases had the CC genotype for rs13095226 in the population-based control group or HM without CNV. Thus, multivariate logistic regression analysis of this genetic region could not be conducted. However, allele analysis showed significant differences in the C allele of SNP rs13095226 in mCNV patients vs the HMnoCNV group, and further studies with a larger sample size are needed to replicate these genes and other candidate loci for this important vision-threatening complication of HM. The second limitation is that the groups showed demographic differences particularly in terms of age, but these subjects were included to obtain a population-representative control group with lower expectations of having CNV. Furthermore, to minimize the possible influence of these differences, we adjusted for all confounding factors using logistic regression analysis and found no new significant associations.

In summary, the results of this study showed that age and HT were significantly associated with CNV development in highly myopic Caucasian patients and that *COL8A1* plays an important role in axial elongation of the eye and possibly in the CNV. The function of *COL8A1* in remodeling of the extracellular matrix of the sclera may involve genetic pathways in the development of CNV in highly myopic eyes in elderly Caucasian populations. Furthermore, additional studies are needed to determine whether any of the SNPs analyzed are associated with the size of the CNV.

## Supporting Information

S1 Table Characteristics of the Candidate SNPs genotyped. SNP: Single nucleotide polymorphism (dbSNP1D); MAF: Minor allele frequency, HWE: exact test for Hardy-Weinberg equilibrium. *Excluded from analysis because all patients showed CC genotype.
(DOCX)

S2 Table Meta-analysis results of three SNPs (rs669676, rs10033900 and rs10468017) in mCNV. OR; odds Ratio, D+L pooled OR; random effect model OR, I-V pooled ES; fixed effect model OR, d.f.; degree of freedom.
(DOCX)
