# Supplementary material for: Evaluation of 10 AMD Associated Polymorphisms as a Cause of Choroidal Neovascularization in Highly Myopic Eyes
Source: PLoS One. 2016 Sep 19;11(9):e0162296. doi: 10.1371/journal.pone.0162296 (PMC5028023; doi:10.1371/journal.pone.0162296)
Supplement: S1 Table — SNP: Single nucleotide polymorphism (dbSNP1D); MAF: Minor allele frequency, HWE: exact test for Hardy-Weinberg equilibrium. *Excluded from analysis because all patients showed CC genotype. (DOCX) [file pone.0162296.s001.docx]

**S1 Table Characteristics of the Candidate SNPs genotyped**

| dbSNP ID | Gene | Chromosome region | Applied Biosystems | Major/Minor  Allele | HWE |
| --- | --- | --- | --- | --- | --- |
| rs13095226 | *Col8A1* | 3q12.1b | C_26159211_10 | T/C | 0.0087 |
| rs669676 | *Col8A1* | 3q12.1c | C_819292_10 | G/A | 0.14 |
| *rs769455 | *APOE* | 19q13.32a | C_3084794_10 | C/T | - |
| rs10033900 | *CFI* | 4q25c | C_34681305_10 | C/T | 1 |
| rs11728699 | *CFI* | 4q25c | C_2551834_10 | T/G | 1 |
| rs13117504 | *CFI* | 4q25c | C_1804635_10 | C/G | 0.9 |
| rs11726949 | *CFI* | 4q25c | C_32217579_10 | C/T | 0.53 |
| rs6854876 | *CFI* | 4q25c | C_29422910_10 | G/C | 0.8 |
| rs7439493 | *CFI* | 4q25c | C_32217575_10 | G/A | 0.8 |
| rs10468017 | *LIPC* | 15q21.3d | C_29910029_10 | C/T | 0.0065 |

SNP: Single nucleotide polymorphism (dbSNP1D); MAF: Minor allele frequency, HWE: exact test for Hardy-Weinberg equilibrium.

*Excluded from analysis because all patients showed CC genotype
