# Supplementary material for: Evaluation of 10 AMD Associated Polymorphisms as a Cause of Choroidal Neovascularization in Highly Myopic Eyes
Source: PLoS One. 2016 Sep 19;11(9):e0162296. doi: 10.1371/journal.pone.0162296 (PMC5028023; doi:10.1371/journal.pone.0162296)
Supplement: S2 Table — OR; odds Ratio, D+L pooled OR; random effect model OR, I-V pooled ES; fixed effect model OR, d.f.; degree of freedom. (DOCX) [file pone.0162296.s002.docx]

| **rs669676** | | | |
| --- | --- | --- | --- |
| **Study** | ES | [95% CI] | % Weight |
| **Leveziel et al.** | 1.9 | 1.2-3.0 | 53.7 |
| **Velazquez-Villoria et al.** | 1.1 | 0.7-1.8 | 46.3 |
| **I-V pooled ES** | 1.467 | 1.0-2.1 | 100.00 |
| **Heterogeneity chi-squared = 2.38** | **(d.f. = 1) p = 0.123** | | |
| **I-squared = 58.0%** | **Test of ES=1 : z= 2.21 p = 0.027** | | |
| **rs10033900** | | | |
| **Study** | ES | [95% CI] | % Weight |
| **Leveziel et al.** | 2.1 | 1.2-3.5 | 28.54 |
| **Miyake et al.** | 1.0 | 0.9-1.2 | 45.10 |
| **Velazquez-Villoria et al.** | 1.3 | 0.7-2.3 | 26.36 |
| **D+L pooled ES** | 1.348 | 0.9-2.3 | 100.00 |
| **Heterogeneity chi-squared = 6.43** | **(d.f. = 2) p = 0.040** | | |
| **I-squared = 68.9%; Tau-squared = 0.10** | **Test of ES=1 : z= 1.35 p = 0.176** | | |
| **rs10468017** | | | |
| **Study** | ES | [95% CI] | % Weight |
| **Leveziel et al.** | 0.8 | 0.5-1.4 | 55.54 |
| **Velazquez-Villoria et al.** | 1.1 | 0.6-2.0 | 44.46 |
| **I-V pooled ES** | 0.928 | 0.6-1.4 | 100.00 |
| **Heterogeneity chi-squared = 0.55** | **(d.f. = 1) p = 0.458** | | |
| **I-squared = 0.0%** | **Test of ES=1 : z= 0.36 p = 0.715** | | |
